# Cryopreserved Mesenchymal Stem Cells Stimulate Regeneration in an Intervertebral Disc

**DOI:** 10.3390/biomedicines3030237

**Published:** 2015-08-07

**Authors:** Nataliia Volkova, Mariia Yukhta, Anatoliy Goltsev

**Affiliations:** Laboratory of Biotechnology and Applied Nanotechnology, Department of Cryobiology of Reproductive System, Institute for Problems of Cryobiology and Сryomedicine of the National Academy of Sciences of Ukraine, Kharkov 61015, Ukraine; E-Mails: volkovanatali2006@yandex.ua (N.V.); cryo@online.kharkov.ua (A.G.)

**Keywords:** cell therapy, disease modeling, intervertebral disc, mesenchymal stem cells, regenerative medicine

## Abstract

Background: Degenerative diseases are a medical, social, and economic problem worldwide. The most significant factors predisposing the development of degenerative changes in intervertebral discs are a low density and poor biosynthetic potential of the cells. Therefore, stem cell therapy in this case should show high clinical efficiency. Methods: The research aim was to evaluate the regenerative potential of cryopreserved mesenchymal stem cells (MSCs) upon degenerative changes in intervertebral discs. Rats with simulated degenerative damage of the intervertebral disc Co6–Co7 were administrated with 0.5 × 10^6^ of either native or cryopreserved cells on a collagen sponge to the defect area. The results of experiments were histomorphometrically evaluated on the 30th, 60th, and 90th days after treatment. Results: The restoration of tears, clefts, and collagen fiber fragmentations was noted on the 60th and 90th day after administration of native and cryopreserved MSCs respectively. An increase in fibrochondrocyte density got ahead of the annulus fibrosus height recovery. In the control group without treatment the regeneration was hardly observed. Conclusion: The use of MSCs promotes the restoration of the degenerated intervertebral disc. Cryopreserved MSCs have a “lag” therapeutic effect at the early stages, but show similar results to the native analogue on the 90th day after administration.

## 1. Introduction

An intervertebral disc (IVD) is the second most important structural formation of the spine and, therefore, its lesions are sure to affect the function of the whole vertebral column. At the same time, the reparative ability of IVD is quite low because of the small numbers of resident cells capable of proliferation. For example, cellular components of the annulus fibrosus (AF) are only 1% of the total tissue weight. Accordingly, the decrease in cell density with age or due to a pathology leads to incomplete recovery and degenerative changes [1].

Another feature of IVDs, which also induce an early development of degenerative changes, is a low intensity of metabolic processes. For many organisms, oxidative reactions in the AF and nucleus pulposus (NP) occur mainly anaerobically. For example, 99% of oxidative reactions in NP cells are anaerobic, and only 1% is aerobic respiration. On average, the oxidative reactions in cartilaginous tissue are at least 50 times less intense than those in other tissues. However, the nature of anaerobic oxidation in chondrocytes is a protective and adaptive response determined by the avascular structure of IVDs [2].

Thus, the most significant factors predisposing IVD degeneration are a low density and the low biosynthetic potential of the cells [3]. From the above, it can be assumed that cell therapy of dorsopathies should show a high clinical efficiency. It is known that IVDs are formed as a result of the interaction between mesenchymal and notochordal cell types, so an alternative source for its repair can be mesenchymal stem cells (MSCs) from different sources that have been successfully used in orthopedics and traumatology. MSCs are adult tissue-derived progenitor cells that exhibit clonogenicity, extended proliferative activity, and the ability to undergo multi-lineage differentiation [4]. Some *in vitro* studies showed that MSCs under certain conditions were able to differentiate into AF [5] and NP cells [6].

The use of modern technology of cultivation and cryopreservation promotes the obtaining of stem cell reserves and their long-term storage at low temperatures without significant changes in morphological and functional states. This allows the free transport of cells and their thawing just before therapeutic use. Hence, the estimation of efficiency and safety of cryopreserved MSCs upon degenerative changes in the IVD is an actual problem.

Sometimes the method of cell administration can be a limiting factor of therapeutic efficiency. Nowadays, there are several approaches to cell-based therapy for degenerated IVDs, the basis of most of which is an injection. In our view, it is not the most appropriate route of administration because it can enhance the IVD damage [7]. The authors of [8] also claim that the size of the AF tears and clefts is not correlated with the location and size of the puncture needle and, in all cases, deep damage beyond self-repair was formed due to the low metabolic activity of fibrochondrocytes.

Based on this, the aim of this study was to evaluate the regenerative potential of native and cryopreserved bone marrow MSC administration to the degenerated IVD.

## 2. Experimental Section

### 2.1. Animals and Ethics Statement

The experiments were performed on 98 male rats weighing 325 ± 25 g (mean ± standard deviation). All the rats were housed in plastic cages (one animal per cage) and kept at a controlled temperature (18–22 °C), humidity (30%–70%), and lighting (lights on from 8 a.m. to 8 p.m.) on a standard diet with free access to food and water. Rats were acclimated for at least seven days prior to experimentation. During housing, animals were monitored daily for health status. No adverse events were observed. All manipulations were carried out in strict accordance with the requirements of the “European Convention for the Protection of Vertebrate Animals used for Experimental and other Scientific Purposes”. The protocol was approved by the Committee on the Ethics of Animal Experiments of the Institute for Problems of Cryobiology and Сryomedicine of the National Academy of Sciences of Ukraine (Permit No 2014-02). A completed ARRIVE guidelines checklist is included in Checklist S1.

### 2.2. Isolation and Culture of MSCs

MSCs were isolated from resected femur of rats (*n* = 7, weighing 100–150 g) by washing out with Hanks’ solution (PAA, Pasching, Austria), followed by flushing through a needle with gradually decreased diameter. The next step was centrifugation at 834× *g* for 5 min. The cells were resuspended in culture medium and plated on culture flasks (PAA) with 10^3^ cells per cm^2^ density. Cultural medium contained: Iskove’s Modified Dulbecco’s Medium (PAA), 10% fetal bovine serum (FBS) (HyClone, Logan, UT, USA), gentamicin (150 mg/mL) (Farmak, Kiev, Ukraine), and amphotericin B (10 mg/mL) (PAA). Cultural medium was changed every three days. We used standard culture conditions (37 °C, 5% CO_2_, 95% humidity) in a CO_2_ incubator (Sanyo, Osaka, Japan). Cells were cultured until 75% confluent and then were used either for therapy (native MSCs) or for cryopreservation.

### 2.3. Cryopreservation of MSCs

Cryopreservation solution was the growth medium supplemented with 10% DMSO (PanEco, Moskow, Russia) and 20% FBS. Freezing was performed at 1 °C/min to −80 °C, followed by plunging into liquid nitrogen [9,10]. All samples were stored in a low temperature bank for three months and thawed in a water bath at 40 °C to a liquid phase. Cryoprotectant was removed by slowly adding a 10-fold volume of Hanks’ solution (PAA) followed by centrifugation at 834× *g* for 5 min. The percentage of viable cells was assessed with a trypan blue exclusion test [9]. After being thawed, cells were immediately used for therapy (cryopreserved MSCs).

### 2.4. Animal Model of IVD Degenerative Damage and Treatment

**Figure 1:**
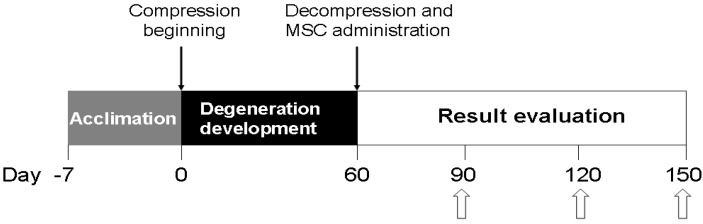
Experimental schedule. White arrows denote time points at which histomorphometric analysis of the AF tissue was done.

Choosing a simulation method, we decided to use the compressive model of IVD degeneration in rats [11] because it is the closest to the pathology observed in humans by mechanisms of damage as well as in the complex of developing histological changes [12]. It was simulated on 91 rats in the following way: the resection of 2/5 length of the tail at the level of Co20–Co21 was carried out and the formed stump was stitched subcutaneously on the back, 1 cm cranially to lumbosacral joint. On the 60th day the compression was stopped, and the animals were randomized into experimental groups: N1 (*n* = 21) with 0.1 mL saline administration on a 0.8 × 0.8 × 0.5 cm collagen sponge (Ankerpharm, Berlin, Germany), which was located in the soft tissue bed in the proximity to the dorsal part of the IVD Co6–Co7; N2 (*n* = 21) with 0.5 × 10^6^ viable native MSC administration on collagen sponge in 0.1 mL of Hanks’ solution; N3 (*n* = 21) with 0.5 × 10^6^ viable cryopreserved MSC administration on collagen sponge in the same volume of Hanks’ solution. In the animals of the control group (*n* = 21) no treatment was applied. The experimenter was blinded to the treatment groups. For all surgical manipulations the animals were anesthetized by an intraperitonial injection of ketamine (10 mg/kg, Biolik, Kharkov, Ukraine) combined with xylazine (1 mg/kg, Bioveta, Prague, Czech Republic). During five days of the postoperative period, animals received Brovaseptol (4 g/kg, Brovafarma, Brovary, Ukraine) of feed to prevent septic complications and ketofen (2 mg/kg, Merial SAS, Lyon, France) for the purpose of anesthesia. Humane euthanasia was performed by CO_2_ asphyxiation on the 30th, 60th, and 90th days after treatment (Figure 1).

In addition, seven animals of corresponding age and weight were used in the experiment for benchmarking (intact rats), and seven animals were used for studying the model on the 60th day of IVD compression, *i.e.*, at the time of the therapy (model indices).

### 2.5. Histomorphometric Analysis

Parts of the spine were taken for histology at level Co4–Co10 and were coded by a third person who was not involved in the experiment to maintain blinding. Celloidin sections of the spine were stained with hematoxylin and eosin, toluidine blue and by van Gieson’s method. The height and fibrochondrocyte density of the dorsal AF of the IVD Co6–Co7 were evaluated by light microscopy (Carl Zeiss, Oberkochen, Germany). The height was determined by the AF terminal elements at the surfaces of the vertebra epiphyses nearest to each other, located above and below. The fibrochondrocyte density was determined as the average of the number of nuclei in the external, central, and internal parts of the dorsal AF with following conversion per 1 mm^2^. For comprehensive assessment of qualitative histological changes of the AF tissue, an integrated scale was developed based on the reports [13,14,15,16] (see Table 1). The degree of collagen fiber fragmentation, tears/clefts, and areas of low density/lack of fibrochondrocytes was estimated by the specific area occupied by pathological elements. In this case, the minimal total number of points (7–8 points) corresponded to the intact rats. A score from 9 to 16 points was accepted as mild degenerative changes of AF tissue, from 17 to 22 points as moderately expressed ones, and in the case of 23 or more points, changes were considered pronounced.

**Table 1:** Integrated semi-quantitative histological analysis of the AF tissue.

Criteria	Grading, Number of Points
*The course of the collagen fiber bundles*	1 = discrete bundles with radial orientation
	2 = serpentine bundles
	3 = reverse bundles
	4 = disorganized bundles
*Collagen fiber fragmentation*	1 = absent
	2 = less than 30% of the AF area
	3 = 30%–60% of the AF area
	4 = more than 60% of the AF area
*Tears/clefts*	1 = absent
	2 = less than 30% of the AF area
	3 = 30%–60% of the AF area
	4 = more than 60% of the AF area
*Hypo-/acellularity*	1 = absent
	2 = hypocellularty
	3 = hypocellularity, acellular areas not more than 30%
	4 = acellular areas more than 30%
*AF/NP boundary*	1 = clear and continuous
	2 = minimally interrupted
	3 = indistinct and interrupted
	4 = NP protrusion with lysed notohordal cells
*Fuchsinophil staining*	1 = collagen fibers are fuchsinophil
	2 = insignificant reduction
	3 = significant reduction
	4 = collagen fibers are picrinophil
*State of the adjacent vertebrae apophyses*	1 = vertebral edges are rounded
	2 = vertebral edges are sharpened
	3 = chondrophytes, early osteo-phytes
	4 = pronounced osteophytes

In addition, the cell size and nucleus shape were taken into account when studying the cell population of AF tissue. If a nucleus length was equal to its width, the nucleus was considered spherical. If the length-to-width ratio was in the range from 1 to 2, the nucleus was considered ovoid. A nucleus with the ratio >2 was considered spindle-shaped.

### 2.6 Statistical Analysis

The results were processed with a non-parametric Mann-Whitney *U*-test using “Statistica 8”. The results were presented as mean ± standard deviation. *p* < 0.05 was considered statistically significant.

## 3. Results

**Figure 2:**
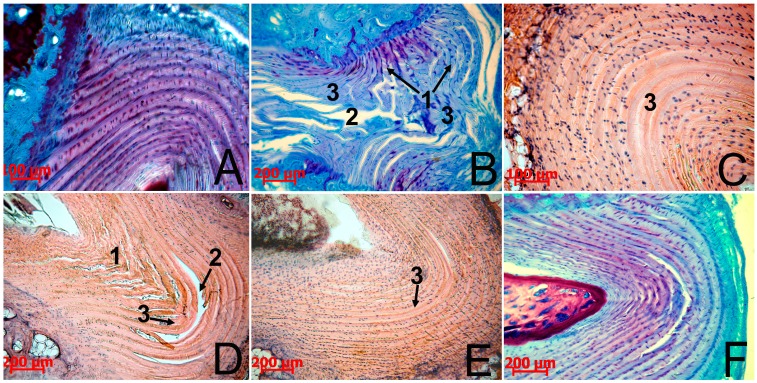
The IVD Co6–Co7 in rats. Collagen fiber fragmentation (1), central tears and clefts (2), and areas with low fibrochondrocyte density or complete absence of cells (3) were observed in the AF of animals without treatment (**B**) even on the 90th day. In this group, metachromatic staining similar to the intact animals (**A**) was absent. The decrease in collagen fiber fragmentation and the significant reduction in the area occupied by tears, clefts, and acellular sites were observed in the AF of animals after native MSC therapy on the 30th day (**C**) and cryopreserved MSC therapy on the 30th (**D**) and 60th (**E**) days. On the 90th day, after cryopreserved MSC administration (**F**), almost complete recovery of the AF histological structure and its metachromatic staining was observed.

**Figure 3:**
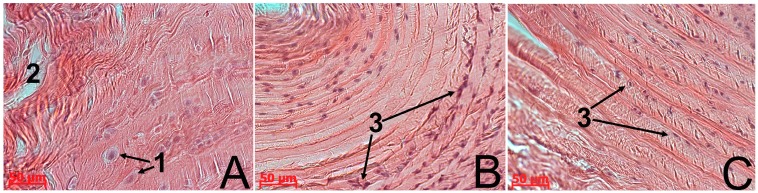
The IVD Co6–Co7 in rats. In the control group of animals (**A**), large chondrocyte-like cells with spherical nuclei surrounded by a wide rim of cytoplasm (1) were detected near the clefts (2). In the case of native MSC administration (**B**) as well as the cryopreserved one (**C**), the AF cell population was represented by small fibrochondrocyte-like cells with ovoid nuclei (3).

The results of the morphological study showed that restoration in the control and experimental group N1 was delayed, with insufficient synthesis of regenerative products. This was probably due to the failure of the body’s internal resources and the inability of the AF tissue to attain full recovery. Despite the cessation of compression in these groups, little spontaneous normalization, if at all, of all the test parameters occurred. At all the observation stages there was significant histological evidence of the degenerative damage of the AF tissue (Figure 2B): collagen fiber fragmentation, central tears and clefts, areas with low fibrochondrocyte density or a complete absence of cells, indistinct and interrupted AF/NP boundaries. AF cells either located singly or formed isogenic groups and their structure was heterogeneous (Figure 3A). Along with fibrocartilage-specific cells with ovoid nuclei, there were large chondrocyte-like cells with spherical nuclei surrounded by a wide rim of cytoplasm, which were located close to clefts. This fibrochondrocyte transformation with a strongly pronounced decline in cell density indicates the tension of regenerative processes in this area of AF [1]. At the same time, it should be noted that the method of the local MSC administration on the collagen sponge did not have a negative impact on the IVD structure.

Microscopic studies of the IVD histological sections showed a partial regeneration of the AF structure on the 30th day after native MSC administration (Figure 2C). The restoration of tears, clefts, and collagen fiber fragmentations was observed and the AF/NP boundary became more distinct. Fibrobchondrocyte-like cells with dense ovoid nuclei were located not only along collagen fiber bundles, but also thickly penetrated into their mass, indicating a high rate of reparative processes in the IVD. Cell density on the dorsal side (the side of MSC administration) was significantly higher in comparison with the opposite (ventral) part of the AF, although the central areas of the dorsal side in this period of observation remained acellular, and bundles of collagen fibers were occasionally slightly fragmented.

Cryopreserved MSCs also stimulated the AF tissue regeneration in the IVD Co6–Co7, but the proliferative response of fibrochondrocytes was less pronounced compared to the native analogue. There was a tendency for improvement in the AF histological structure of the IVD Co6–Co7 on the 30th day (Figure 2D), namely with a decrease in collagen fiber fragmentation, a significant reduction in the area occupied by tears, clefts, and acellular sites, and an improvement of the AF/NP boundary contours. The population of AF cells, as well as in the case of native MSC administration, was represented by small fibrobrochondrocyte-like cells with ovoid nuclei, but cells were located mainly along collagen fibers, only penetrating their mass in some areas (Figure 3B,C).

On the 60th day after native MSC administration, almost complete recovery of the IVD Co6–Co7 histological structure was observed, except for an insignificant collagen fiber fragmentation. The cryopreserved MSC administration was accompanied by the progression of regenerative changes in the IVD Co6–Co7, although quite pronounced degenerative signs (small areas with low density or complete absence of fibrochondrocytes surrounding the centrally located cleft) persisted (Figure 2E).

Regeneration of the IVD structure after cryopreserved MSC administration only occurred on the 90th day after treatment (Figure 2F). In this case, the IVD histology was similar to that on the 60th day after native MSC administration.

The integrated assessment of histological changes (Table 2) showed that in the animals of the control and experimental group N1, the total score at all the observation stages was almost unchanged and corresponded to the pronounced degenerative changes. In cell-treated animals there was a gradual improvement in the histological structure from moderately expressed degenerative changes on the 30th day to mild ones on the 60th day, with recovery on the 90th day after the administration both of native and cryopreserved MSCs.

**Table 2:** Integrated semi-quantitative histological analysis of the AF tissue after MSC transplantation (the total score). The limit values of the parameter for the corresponding group of animals are shown in parentheses.

Group of Animals (*n* = 7)	Study Day, Number of Points		
	30	60	90
Control group (no treatment)	24.3 (23–26)	23.7 (22–25)	23.3 (22–25)
Experimental group N1 (saline administration)	23.9 (23–26)	24.1 (23–25)	23.7 (22–25)
Experimental group N2 (native MSC therapy)	16.3 (13–19)	8.0 (7–9)	7.3 (7–8)
Experimental group N3 (cryopreserved MSC therapy)	19.4 (16–22)	14.0 (11–18)	7.9 (7–9)
Intact animals	7.6 (7–9)		

**Figure 4:**
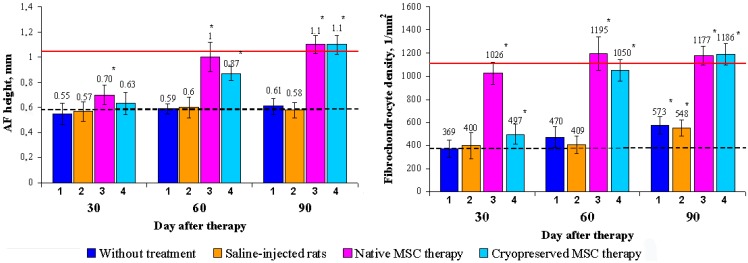
Dynamics of the AF height and fibrochondrocyte density restoration in rats. AF height and fibrochondrocyte density were significantly elevated in the native MSC therapy group (3) compared to model indices (dotted line) throughout the observation period and reached the values of intact animals (solid line) on the 30th and 60th days, respectively. In the cryopreserved MSC therapy group (4), a significant elevation in the AF height was observed only from the 60th day on, whereas the fibrochondrocyte density significantly differed compared to the model index (dotted line) throughout the observation period. In animals without treatment (1) and saline-injected rats (2), the fibrochondrocyte density was only slightly elevated on the 90th day, but did not reach the values of intact animals (solid line). * Statistical significance compared with the model index (*p* < 0.05; *n* = 7).

For an objective assessment of the identified qualitative changes, the height and fibrochondrocyte density were studied in the dorsal part of the AF (Figure 4). The results showed that the AF height on the 30th day after cryopreserved MSC administration increased (12.5% ± 3.3%) related to the model values. The fibrochondrocyte density increased as well (26.1% ± 2.9%). Accordingly, on the 30th day, the group with the administration of native MSCs was accompanied by an increase of fibrochondrocyte density (3.5-fold) and AF height (25.3% ± 4.1%). It should be noted that in both groups of MSC-treated animals, the fibrochondrocyte density got ahead of the AF height recovery. In the control and experimental group N1 a tendency to restoration was hardly observed (Figure 4).

## 4. Discussion

By this time, publications revealed that depending on the degree of degenerative changes in IVDs, MSC transplantation may have an inhibitory or blocking effect on the further progression of the pathological process without pronounced reparative changes in the IVD [17]. However, it should be noted that in these studies, MSCs were administered by intra-disc injections that might hinder regeneration, causing a negative impact on mechanotransduction and stimulating degenerative changes in the AF tissue due to the low metabolic activity of fibrochondrocytes [3].

In this study we observed that local administration of cryopreserved MSCs on a collagen sponge had a stimulating effect on the regenerative processes in degenerated AF tissue, qualitatively affecting their course. In the control group, the dominant component of the regeneration in AF was synthetic, and in the cell-treated groups, the proliferative component was prevalent. The MSC therapy provided the most pronounced stimulatory effect on the fibrochondrocyte density and a lesser-expressed effect on the AF height of the IVD Co6–Co7. This may be due to indirect correlation between these parameters and requires further studies with a long-term follow-up.

Previous studies have illustrated that cartilage regeneration depends on the modulating action of certain hormones. For example, corticosteroids inhibit anabolic reactions, collagen and proteoglycan synthesis in chondrocytes, and cause hyaluronic acid deficiency in the intercellular matrix. This inhibitory effect of glucocorticoids is more pronounced if it is combined with cartilage compression [18]. This is generally not surprising, when one takes into account that glucocorticoids inhibit glycolysis, *i.e.*, the anaerobic oxidation of glucose in cartilage. Regeneration without an energy supply becomes impossible. Consistent with our hypothesis, the recovery effect of MSCs, including cryopreserved ones, on the degenerative damage of AF tissue, as in the case of articular cartilage, is attributed to the paracrine and mitotic activity of the transplanted cells. MSCs are known to synthesize a wide range of cytokines and chemokines, including some of the most potent anti-apoptotic, anabolic, and mitogenic stimulants of cartilage regeneration such as insulin-like growth factor-1 and transforming growth factor β [19,20], which dramatically increase the proliferative and synthetic activities of fibrochondrocytes [21].

In our work we also noted that the restoration of AF tissue occurred through an increase in cellularity in outer parts of the AF and its areas adjacent to the vertebra apophyses, which is generally in line with the principles of articular cartilage regeneration.

## 5. Conclusions

This study demonstrated that MSCs contributed to the recovery of the degenerated IVD, although the comparison of cryopreserved MSCs with the native analogue demonstrated a “lag” therapeutic effect at the early study terms. The result of native MSC administration was similar to that of the cryopreserved administration, but at a later term (on the 90th day).

Thus, we experimentally showed the possibility of cryopreserved bone marrow MSC administration on a collagen sponge for the restoration of degenerated AF tissue, which is the first step in confirming the effectiveness of their use in future clinical trials. Although only one cell population has been used, our results suggest that this novel methodology has major implications for the future of cell-based, tissue engineering strategies for the treatment of the degenerated IVD because it allows rapid production of fibrochondrocyte-like cells without the dangers of IVD tissue damage.
